# HematoPorphyrin Monomethyl Ether polymer contrast agent for ultrasound/photoacoustic dual-modality imaging-guided synergistic high intensity focused ultrasound (HIFU) therapy

**DOI:** 10.1038/srep31833

**Published:** 2016-08-18

**Authors:** Sijing Yan, Min LU, Xiaoya Ding, Fei Chen, Xuemei He, Chunyan Xu, Hang Zhou, Qi Wang, Lan Hao, Jianzhong Zou

**Affiliations:** 1 State Key Laboratory of Ultrasound Engineering in Medicine Co-Founded by Chongqing and the Ministry of Science and Technology, Chongqing Key Laboratory of Biomedical Engineering, College of Biomedical Engineering, Chongqing Medical University, Chongqing 400016, P. R. China; 2 Ultrasound department of Second Hospital Affiliated to Chongqing Medical University, Institute of Ultrasound Imaging, Chongqing Medical University, Chongqing 400010, P. R. China; 3 Ultrasound department of First Hospital Affiliated to Chongqing Medical University, Chongqing 400016, P. R. China; 4 Second Affiliated Hospital of Chongqing Medical University & Chongqing Key Laboratory of Ultrasound Molecular Imaging, 400016, P. R. China

## Abstract

This study is to prepare a hematoporphyrin monomethyl ether (HMME)-loaded poly(lactic-co-glycolic acid) (PLGA) microcapsules (HMME/PLGA), which could not only function as efficient contrast agent for ultrasound (US)/photoacoustic (PA) imaging, but also as a synergistic agent for high intensity focused ultrasound (HIFU) ablation. Sonosensitizer HMME nanoparticles were integrated into PLGA microcapsules with the double emulsion evaporation method. After characterization, the cell-killing and cell proliferation-inhibiting effects of HMME/PLGA microcapsules on ovarian cancer SKOV_3_ cells were assessed. The US/PA imaging-enhancing effects and synergistic effects on HIFU were evaluated both *in vitro* and *in vivo*. HMME/PLGA microcapsules were highly dispersed with well-defined spherical morphology (357 ± 0.72 nm in diameter, PDI = 0.932). Encapsulation efficiency and drug-loading efficiency were 58.33 ± 0.95% and 4.73 ± 0.15%, respectively. The HMME/PLGA microcapsules remarkably killed the SKOV_3_ cells and inhibited the cell proliferation, significantly enhanced the US/PA imaging results and greatly enhanced the HIFU ablation effects on ovarian cancer in nude mice by the HMME-mediated sono-dynamic chemistry therapy (SDT). HMME/PLGA microcapsules represent a potential multifunctional contrast agent for HIFU diagnosis and treatment, which might provide a novel strategy for the highly efficient imaging-guided non-invasive HIFU synergistic therapy for cancers by SDT in clinic.

In recent years, rapidly developed high intensity focused ultrasound (HIFU) has been regarded to be a new technique for non-invasive ablation of local tumors, focusing low-energy ultrasound (US) on the target areas in a certain way, which treats the lesion via cell necrosis^1^ caused by the instant heat effect, cavitation effect, mechanical effect, and so on. Over the past few decades, HIFU has gained wide recognition for its safety and efficiency in treating solid tumors and non-tumor diseases^2^,^3^,^4^. However, the therapeutic efficiency of HIFU is relatively unsatisfactory for large and/or deep lesions, due to the exponentially attenuated US energy with increased depth and the reduced energy for the areas adjacent to high-speed blood flow^5^,^6^. Therefore, increased output power and extended treatment duration are always required, which would cause damages to the normal tissue along the US propagation path, leading to severe side effects^7^,^8^,^9^. For these reasons, synergistic agents have emerged to maintain the advantage of non-invasive HIFU treatment, which increase the energy deposition in the target area by affecting the acoustic environment. Ever since the discovery of the microbubble contrast agents with various formulations, numerous studies have been focusing on exploring novel synergistic agents for HIFU^10^. It has been shown that, lipid microbubbles and polymer microspheres can produce a synergistic effect in HIFU ablation, which efficiently enhance the therapeutic outcome of HIFU. However, the application of these lipid microbubbles is limited due to the instability, friability, and short blood circulation time. Moreover, the size of traditional lipid microbubbles (ranging from several to hundreds micrometers) is usually too large to penetrate through the barrier between vascular endothelial cells and tumor cells. On the other hand, inorganic nanoparticles are always difficult to use in clinical practice because of the toxicity, low biocompatibility, and biodegradability.

Sono-dynamic chemistry therapy (SDT), in which US irradiation is combined with sonosensitizers, could cause irreversible damages to tumor cells with US irradiation in certain frequency and intensity to activate the internal accumulated sonosensitizers. Hematoporphyrin monomethyl ether (HMME), a porphyrin sonosensitizer amphiphilic to lipid phase and aqueous phase, is characterized by its single form, high yield of singlet oxygen, high selectivity, and low toxicity, which has been widely used in the diagnosis and treatment of various tumors, including lung cancer, bladder cancer, and nevus flammeus and brain glioma^11^,^12^. However, the hydrophobicity of HMME makes it easy to assemble in water, leading to low bioavailability and light absorption, which limits its application in clinic. On the other hand, poly (lactic-co-glycolic acid) (PLGA) is one of the preferential candidates for drug delivery, molecular imaging, and tissue engineering in clinic, which is featured by high stability, biocompatibility, biodegradability, and long blood circulation time *in vivo*. Due to its excellent acoustic property, PLGA is commonly used in the preparation of US imaging agents^13^,^14^,^15^, which has been accredited by FDA and formally listed as pharmaceutical excipient into the United States Pharmacopeia. Combination of HMME and PLGA would not only overcome the disadvantages of HMME, but also exert synergistic effect to make an ideal SDT agent.

As one of the representative biomedical imaging technologies in the 21^st^ century, photoacoustic (PA) imaging^16^ provides the tomographic or three-dimensional images of biological tissues or materials with the photoacoustic effect. PA imaging could not only achieve high imaging contrast, sensitive to tissue functional properties as optical imaging, but also create high-resolution images of deep tissues as acoustic imaging. Therefore, PA imaging has a good application prospect in the high-resolution imaging of tumor tissues and functions in clinic^17^. Previous studies have shown that, porphyrins can be applied in the photo- and acoustic-dynamic treatment on tumors. In comparison with other contrast agents for PA imaging (such as gold and carbon nanomaterials), porphyrins are associated with less toxic effects, better biocompatibility, and easy structural modification^18^,^19^. Numerous studies have demonstrated that the combination of US and sonosensitizers is able to generate free radicals and reactive oxygen species (ROS) to destroy tumor cells^20^,^21^, exerting potent anti-tumor effect.

Along with the rapid development of molecular imaging technology, imaging contrast agents with single function and single imaging modality could not meet the increasing demands for diversification and personalization in healthcare. In this study, using HMME with excellent imaging and SDT properties together with PLGA as film-forming material with great biocompatibility, a multifunctional microcapsule was established. The microcapsule integrated the inexpensive, real-time US imaging with excellent PA imaging properties, to offer better images for HIFU diagnosis, and to effectively intensify the HIFU treatment as well. Schematic illustration of this article is shown in Fig. 1.

## Results

### Characterization of HMME/PLGA microcapsules

**Figure 2:**
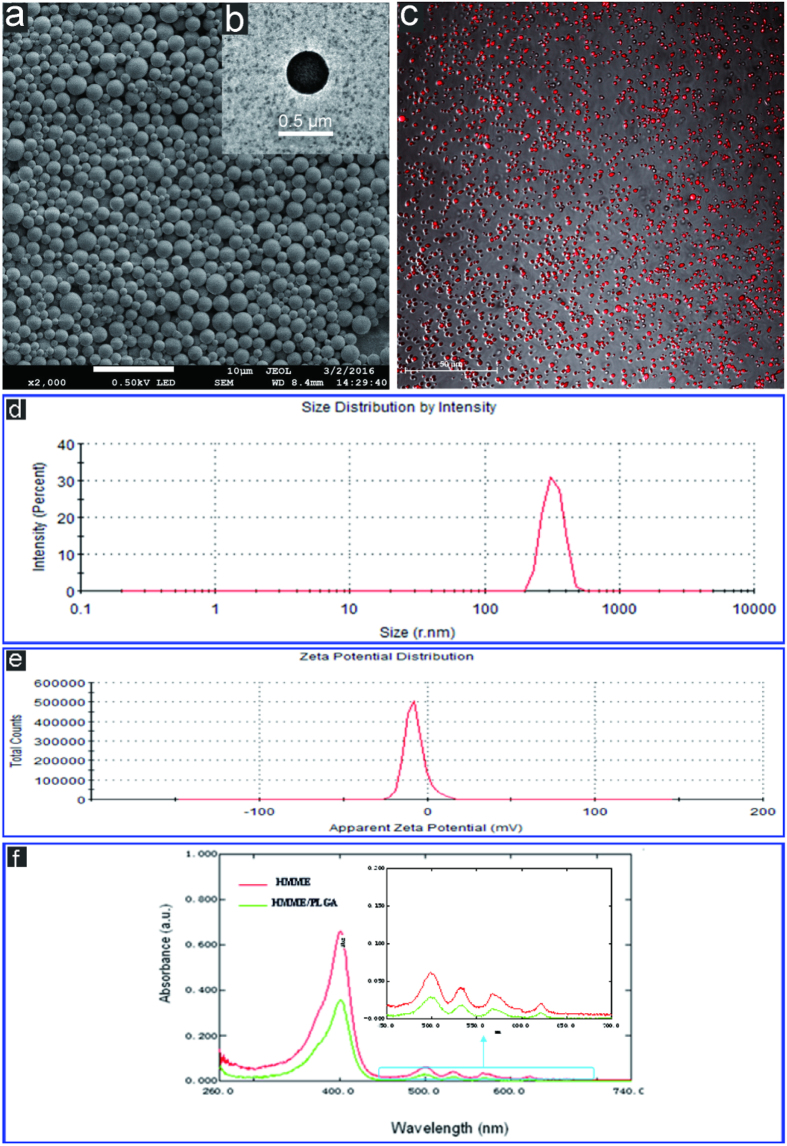
(**a**) SEM image of HMME/PLGA microcapsule. (**b**) TEM image of HMME/PLGA microcapsule. (**c**) CLSM image of HMME/PLGA microcapsule. (**d,e**) Size distributions and zeta potential of HMME/PLGA microcapsule. (**f**) Ultraviolet visible spectrum of HMME and HMME/PLGA microcapsule.

SEM imaging indicated that the microcapsules exhibited a smooth and uniform spherical morphology (Fig. 2a). TEM indicated the presence of HMME in the shells of these microcapsules (Fig. 2b).The HMME/PLGA microcapsules exhibited strong red florescence (Fig. 2c) as detected with CLSM. Moreover, DLS showed that, the average diameter of the HMME/PLGA microcapsules was 357 ± 0.72 nm (PDI = 0.932) (Fig. 2d), and the microcapsule surface was negatively charged (zeta potential: −7.89 mV) (Fig. 2e). Furthermore, UV spectrometry showed that the HMME/PLGA microcapsules covered a wide absorption wavelength range in the UV visible region, with a strong absorption band at 418 nm and four absorption peaks between 500–700 nm (Fig. 2f).

### Encapsulation efficiency and drug-loading efficiency of HMME/PLGA microcapsules

Our results showed that, the encapsulation efficiency of the HMME/PLGA microcapsules was 58.33 ± 0.95%, and the drug-loading efficiency was 4.73 ± 0.15% (w/w).

### HMME/PLGA microcapsules in cell toxicity-proliferation test

**Figure 3:**
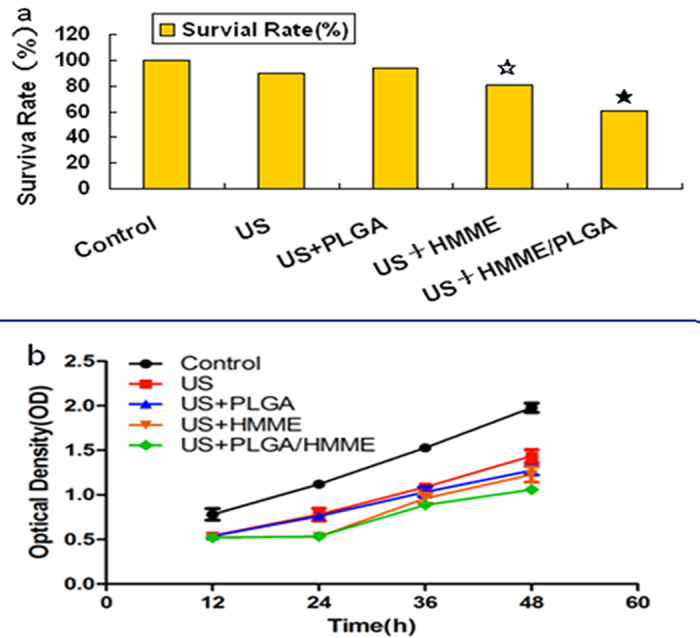
(**a**) The ultrasound irradiation effect of Groups I-V on the survival rate of SKVO_3_ cell at 10 s. ^★^*P *< 0.05 vs the other groups, ^☆^*P *< 0.05 vs US, US+PLGA, and control groups. (**b**) Proliferating activity of SKOV_3_ cells of Groups I-V after the treatment at 12, 24, 36 and 48 h, respectively.

According previously published findings^22^, the cell toxicity and proliferation were assessed with the selected parameters (the US irradiation intensity of 0.50 w/cm^2^ and frequency of 1 MHz). The HMME concentration for Groups IV and V was 20 μg/ml. In the cell toxicity experiment, the cell survival rate was significantly decreased along with the prolonging duration of US irradiation (Table 1). Meanwhile, at the same time points (Fig. 3a), Group I had the highest cell viability, while the cell viability was decreased from Group II to Group V, among which Group V reported the largest reduction, followed by Group IV. Groups II and III had no significant difference, and compared with Group I, the difference was not significant.

Given that US irradiation would inevitably induce damages in normal cells while killing tumor cells, the cell proliferation test was performed with the parameters of 0.50 w/cm^2^ and 10 s. Optical density (OD) reflects the cell proliferation activity. As shown in Fig. 3b, the OD value showed the fastest increasing in Group I, while more slowly increasing OD values were observed for Groups II-V. Among these groups, the slowest OD increasing value was noted for Group V (P < 0.05), followed by Group IV, while Groups II and III did not show any significant difference (P > 0.05).

### *In vitro* and *in vivo* US and PA dual-modality imaging

**Figure 4:**
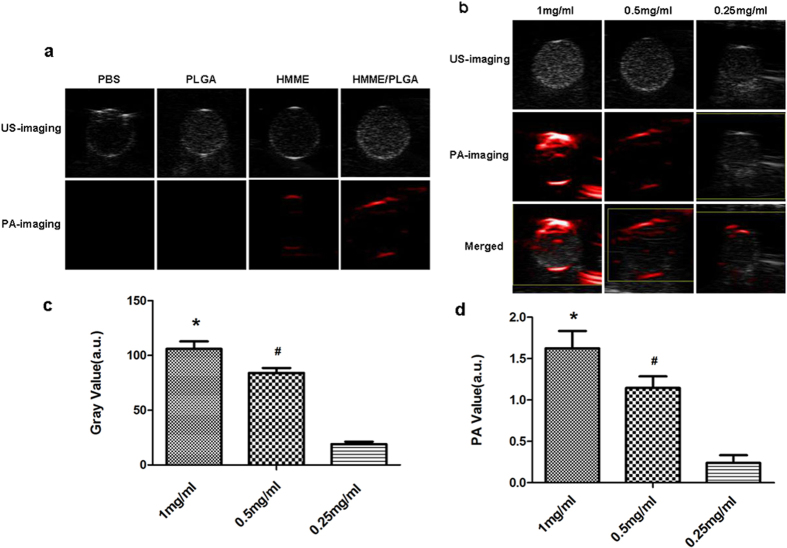
(**a**) *In vitro* US/ PA images of the different samples: PBS, PLGA (0.50 mg/ml), HMME (0.50 mg/ml), HMME/PLGA microcapsules (C_HMME_: 0.50 mg/ml). (**b**) US /PA images and merged images of HMME/PLGA microcapsules with different concentrations of HMME. (**c,d**) The Gray (**c**) and PA (**d**) values of the HMME/PLGA microcapsules with different concentrations of HMME. *P < 0.05 vs the other groups; ^#^*P* < 0.05 vs the 0.25 mg/ml group.

The US imaging signals of PBS, PLGA, HMME, and HMME/PLGA microcapsules were gradually increased, as indicated by the mean echo intensities (mean DB) of the samples. At the same concentration, the HMME/PLGA microcapsules had the strongest echo intensity, while PBS had the weakest. The PLGA and HMME did not exhibit significant difference (Fig. 4a). Meanwhile, the US signals were increased obviously with the increasing HMME concentrations, and the mean echo intensities had significant difference (P < 0.05) (Fig. 4b,c). Next, PA properties of these samples were evaluated. At the same concentration, the PA signal of HMME/PLGA microcapsules was stronger than HMME, while PBS and PLGA almost had no PA signals (Fig. 4a). Moreover, an obvious linear correlation was observed between the HMME/PLGA microcapsule concentration and PA signal (Fig. 4d). Especially, the maximum concentration was associated with the strongest PA signal.

**Figure 5:**
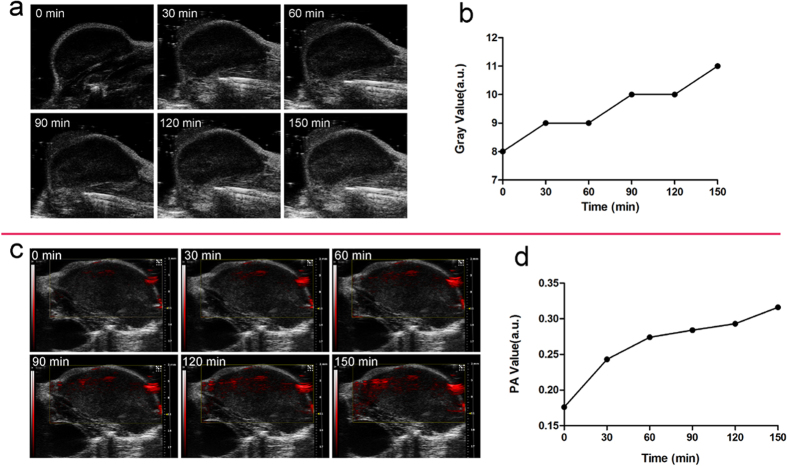
(**a/c**) *In vivo* US/ PA images of tumor tissues at 0, 30, 60, 90,120, and 150 min, respectively, after the injection the HMME/PLGA microcapsules. **(b,d**) The corresponding Gray (**b**) and PA (**d**) values of tumor tissues after the injection of the HMME/PLGA microcapsules.

Based on the outstanding *in vitro* PA/US imaging performance, the *in vivo* imaging performance of HMME/PLGA microcapsules was then investigated. The tumor accumulation of the HMME/PLGA microcapsules (total dose = 0.2 ml, C_HMME_ = 1.5 mg/ml) after systemic intravenous injection was clearly manifested by US/PA imaging. As shown in Fig. 5c,d, the PA signal intensity was gradually increased at the tumor sites for all the time points, while there was very little change in the US signal in the tumor region (Fig. 5a,b).

### *In vitro* and *in vivo* HIFU exposure

**Figure 6:**
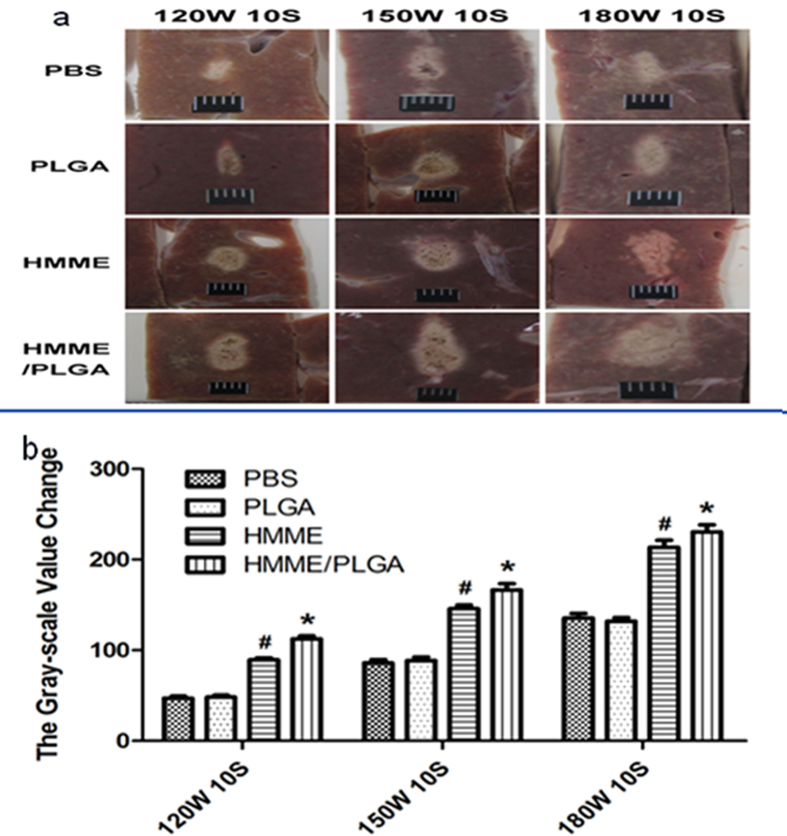
(**a**) The coagulative volume of bovine liver tissue ablated by HIFU with different power (120, 150, and 180 w, respectively) by injection of PBS, PLGA, HMME, and HMME/PLGA, with the exposure duration of 10 s. (**b**) The gray-scale value change of bovine liver tissue by injection of PBS, PLGA, HMME, and HMME/PLGA. *P < 0.05 vs the other groups; ^#^*P* < 0.05 vs PBS and PLGA groups.

High echoes were acquired in the coagulative necrosis tissues after HIFU irradiation. When all the groups were set with the same irradiation parameters (i.e., concentration, acoustic power, and irradiation duration), the coagulative necrosis volume and gray scale variation value in the target area of the HMME/PLGA microcapsule group (Group IV) were increased greater than all the other groups (P < 0.05) (Fig. 6a,b), while the EEF value of Group IV was much smaller than the other groups (see Supplementary Fig. S1). For these indicators, Group III ranked between Groups I and II, and no significant difference was observed between Groups I and II (P > 0.05).

**Figure 7:**
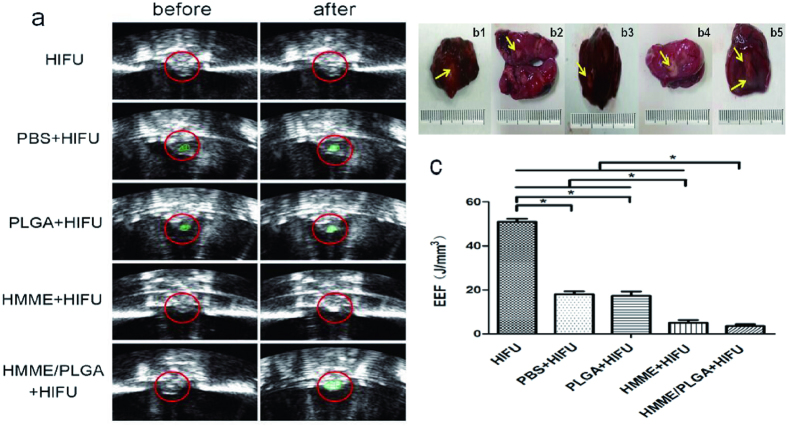
(**a**) *In vivo* ultrasound imaging of tumor tissue (red circles) before/ after HIFU ablation: HIFU, PBS + HIFU, PLGA + HIFU, HMME + HIFU, and HMME/PLGA + HIFU. (**b1–b5**) Ovarian tumors exposed to HIFU after TTC staining (yellow arrows): (**b1**) HIFU, (**b2**) PBS, (**b3**) PLGA, (**b4**) HMME, and (**b5**) HMME/PLGA. The necrotic tissues appeared gray, and the non-ablated tumors were stained red. (**c**) The EEF value of the five groups after HIFU ablation *in vivo*. *P < 0.05.

According to the experimental results and previous studies^23^, the relatively smaller therapeutic power of 120 w and irradiation duration of 5 s were selected. After HIFU irradiation, the tumor tissues in the control group (Group I) had no significant gray scale alteration, while different degrees of gray level changes in tumor were observed for Groups II to V (Fig. 7a). In comparison with Groups II to IV, Group V reported the greatest gray scale change (P < 0.05). Moreover, compared with Groups II and III, Group IV exhibited the greater gray scale change (P < 0.05). No significant difference was observed between Groups II and III (P > 0.05). When the ablation volume was compared among these groups (Fig. 7b1–b5), the greatest coagulative necrosis volume was observed for Group V, followed by Group IV. No significant difference was observed between Groups II and III, and the smallest volume was noted for Group I. The EEF value variation of these groups was similar with the *in vitro* experiment (Fig. 7c).

**Figure 8:**
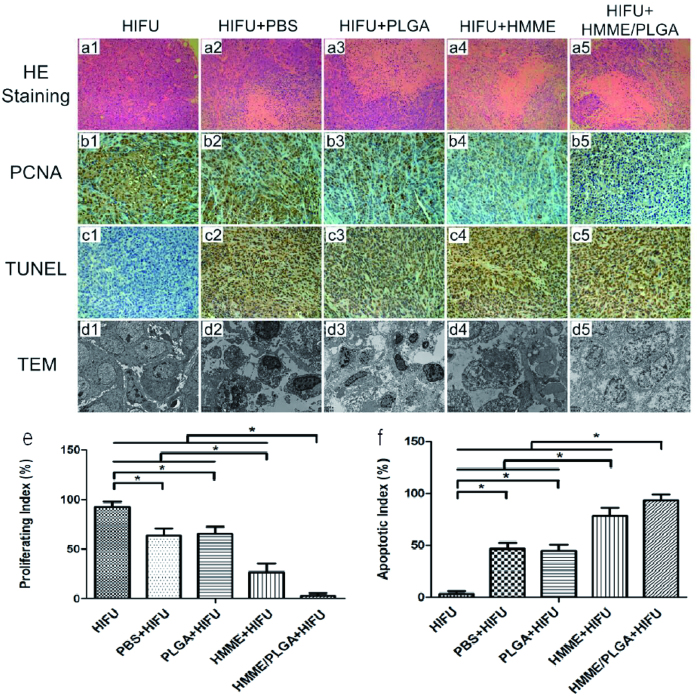
(**a1–a5**) HE staining of the tumor tissues (400× magnification). (**b1–b5**, **c1–c5**) PCNA and TUNEL staining of the tumor tissues (400 × magnification). (**d1–d5**) TEM analysis of the tumor tissues [4000 × (**d1,d3**), 6000 × (**d2,d5**), and 8000 × (**d4**) magnification]. (**e,f**) PI of PCNA and AI of TUNEL in different groups after HIFU ablation. *P < 0.05.

For the microscopic examination, the tumor cells were neatly arranged, with intact cellular morphology in Group I. Severe damages (i.e., nuclear pyknosis, fragmentation, and lysis) were observed, and a mass of substance was stained red uniformly, with scattering cell fragments, in Groups V and IV. The damages in Group IV were slighter than Group V. In the Groups II and III, injuries with various extents were observed (without qualitative differences), and the tumor tissue damages were slighter and weaker than Groups V and IV (Fig. 8a1, 2, 3, 4, 5). Immunohistochemical results displayed that, after the HIFU irritation, the expression of PCNA was reduced or even absent in the necrotic region, while positive expression was observed in the surrounding normal tissues (Fig. 8b1, 2, 3, 4, 5). The results showed that, the PI for the tumors in Group V was significantly lower than the other groups (P < 0.05). Significant differences in PI were observed between these groups, besides Groups II and III (P < 0.05). The tumor tissue expression in Group I was the highest among these groups (P < 0.05) (Fig. 8e). Furthermore, the TUNEL assay was performed to evaluate cellular apoptosis. Similarly to the proliferation experiment, apoptotic cells were observed in all these groups. As shown in Fig. 8c1, 2, 3, 4, 5, the AI for the tumor tissue in Group V was far higher than the other groups (P < 0.05). Significant AI differences were observed between these groups, besides Groups II and III. The tumor tissue expression for Group I was the lowest among all these groups (P < 0.05) (Fig. 8f). As visualized by TEM (Fig. 8d1, 2, 3, 4, 5), in Group I, the cell structures were normal and clear, with intact cell membrane and nuclear membrane and slightly distended mitochondria and endoplasmic reticulum. Cells suffered from the heaviest damages in Group V, where cell structures were destroyed, with broken cell membrane and nuclear membrane, cytoplasm loss, decomposed nucleus substance, and missing organelles. Damages in Group IV were less severe than Group V, and more severe than Groups II and III. Similar damages were observed for Groups II and III, where fractured cell membrane, a small amount of vacuoles in cytoplasm, and swelled mitochondria were observed. The outcomes of TEM were consistent with the HE staining.

## Discussion

SEM and TEM revealed the basic morphology of the HMME/PLGA microcapsules, as a large number of black HMME particles embedded in the PLGA spherical shell. CLSM indicated the HMME/PLGA microcapsules made by ourselves have the potential to be used as fluorescent contrast agents, which will be evidenced in subsequent experiments. DLS demonstrated that most of the microcapsules were on nanometer grade, which is easy to cross the vascular endothelium to enter the tumor tissue. The UV spectrometry suggested that, when embedded in the polymer shell, the absorption spectrum of HMME was not significantly changed^11^. Meanwhile, the absorption spectrum of HMME/PLGA microcapsules provided the basis for the calculation of encapsulation efficiency and drug-loading efficiency, as well as the choice of excitation wavelength in the following PA imaging. The results showed that self-made microcapsules have better encapsulation efficiency and drug-loading efficiency.

In the cell toxicity experiment, the cell survival rate was significantly decreased along with the prolonging duration of US irradiation, indicating that the cell viability had a dose-dependent effect with the US irradiation duration under certain conditions. These results suggested that the HMME/PLGA microcapsules showed the strongest tumor cell-killing effect. The cell proliferation test results indicated that the HMME/PLGA microcapsules performed the best in restraining the proliferation of tumor cells. Compared with HMME alone, the HMME/PLGA microcapsules were more capable of killing tumor cells and inhibiting cell proliferation. One of the reasons might be that, HMME is associated with better dispersion when wrapped in the microcapsule, while HMME alone has worse diffusion, The molecular superposition decreases the molecule activities and consequently declines the sono-dynamic chemistry effect^24^. Cavitation effect^25^ caused by the gas core inside the HMME/PLGA microcapsules might be another reason.

In the *in vitro* experiments, compared with PBS and PLGA, the HMME and HMME/PLGA microcapsules were both capable of US/PA imaging. Compared with HMME alone, HMME/PLGA microcapsules performed better in the imaging. There would be several reasons for this phenomenon. Firstly, HMME is prone to agglomerate in water due to the weak hydrophilicity, which affects its reflectivity to US and absorption of laser energy, while the HMME/PLGA microcapsules have better dispersion, leading to better imaging results. Secondly, the gas core inside the HMME/PLGA microcapsule can increase the backscattering^26^,^27^, thus enhancing the US imaging. Thirdly, as previously reported, polymer shell may play a role in intensifying the US imaging of microcapsules^28^.

The HMME/PLGA microcapsules had excellent PA properties, which could also accumulate in the tumor for a long time, providing the possibility that the HIFU treatment for tumor in a more efficient imaging-guided way. In comparison, the US imaging of HMME/PLGA microcapsules was not so obvious, which may be related to the limited gas cores within the microcapsules. Another explanation might be that, after the intravenous injection of the HMME/PLGA microcapsules into the nude mice, the microcapsule shell would be gradually degraded to induce collapse. Therefore, only limited intact microcapsules may reach the tumor region, while the released HMME would continuously migrate to the tumor region, leading to obvious PA signal while inapparent US signal within the tumor. This would be a limitation of this experiment. Further investigations are still needed to improve the preparation process of these microcapsules.

In the part of the synergism experiment, the synergistic effects of HMME/PLGA microcapsules in the HIFU treatment were investigated, concerning the coagulative necrosis volume, the gray scale variation, and EEF value. Compared with the control group, under the same condition, the HMME/PLGA microcapsules, together with HIFU irradiation, achieved the largest volume of coagulative necrosis in the target area and the greatest gray scale alteration, exhibiting the most potent synergistic effect. This phenomenon could be explained by the following reason: as previously proven, the combination of US and sonosensitizers exerts great anti-tumor effect in SDT. It has been believed by some investigators that the biological function of SDT is based on the cavitation effect^25^,^29^. In the US irradiation, the sonosensitizer (HMME herein) wrapped in the microcapsules were released and effectively activated, which generates singlet oxygen and other substance with strong oxidative resistance^20^,^21^, causing tumor cell death or inhibiting tumor cell growth, and increasing the energy deposition by affecting the acoustic environment. Moreover, other scholars believe that the tumor cell-killing or inhibiting effects of sonosensitizers in SDT are mediated by the thermal effect of US^30^. Tumor tissues are associated with abundant blood supply and slow blood flow. Under US irradiation, HMME/PLGA microcapsules accumulated within the tumor take in the ultrasonic energy to heat up tumors. When the temperature reached a certain level, tumor cells or vascular endothelial cell would suffer from structural and functional damages, which can lead to thrombosis and eventually block the blood vessels, contributing to the deposition of HIFU energy. The examinations showed that the tumor cells in the target area were damaged with different levels, which strongly supported the conclusion above. EEF represents the US energy required to damage the unit volume of tumor tissue. In this study, the smallest EEF value was observed for the HMME/PLGA microcapsule group, indicating that the HMME/PLGA microcapsules would need the least ultrasonic energy to treat unit lesion volume, with the least possible damage to the organism. These findings were consistent to the non-invasive principle of the HIFU treatment, and proved the effectiveness of SDT for the HIFU treatment.

In this study, the HMME/PLGA microcapsules were prepared and used as the contrast agent for efficient US and PA dual-modality imaging. Sonosensitizer HMME was wrapped in the microcapsules as the synergistic agents for HIFU treatment for tumors. The HMME/PLGA microcapsules exhibited excellent contrast-enhanced imaging capability for US and PA dual-modality biological imaging, as demonstrated by the *in vitro* results and the results using *in vivo* tumor models. Importantly, the HMME/PLGA microcapsules had been introduced into the non-invasive HIFU treatment for tumors as the synergistic agent to improve the HIFU therapeutic efficiency. Therefore, administration of microcapsules containing sonosensitizers might be a potential technique to enhance the imaging-guided HIFU treatment for tumors in clinic.

## Methods

### Preparation of the HMME/PLGA nanocapsules

PLGA microcapsules encapsulating HMME (i.e., HMME/PLGA microcapsules) were prepared by the double emulsion (water/oil/water) evaporation method^31^. Briefly, 2 mg HMME (Shanghai D B Chemical Technology Co., Ltd., China) was added into 25 mg PLGA (lactide:glycolide = 50:50; MW = 12000; Daigang, China) dissolved in 2 ml CHCl_3_. After adding 0.2 ml ddH_2_O, the mixture was emulsified using an ultrasonic probe (Sonics & Materials, Inc., USA) at 80 w for 45 s (w/o). Then, the above emulsified solution was poured into 2.5 ml poly(vinyl alcohol) (PVA, MW = 25000, Sigma, USA) solution (5% w/v) and homogenized (FJ300-SH, China) within 45 s for the second emulsion (w/o/w). The final emulsion was mixed mechanically for 2 h to extract CHCl_3_. After centrifugation at 5000 rpm for 5 min, the supernatant was discarded, and the precipitate was washed with ddH_2_O. After centrifugation and washing was repeated three times, the microcapsules were freeze-dried for 48 h, and the dry samples were filled with perfluorocarbon gas (C_3_F_8_) and stored. The freeze-dried power would be used after dissolved in ddH_2_O in the following experiments. Blank PLGA microcapsules were prepared using the same procedures, only in absence of HMME.

### Characterization of HMME/PLGA microcapsules

Microcapsule morphology and structure were characterized with the scanning electron microscopy (SEM, Hitachi S-3400N, Japan) and transmission electron microscopy (TEM, Hitachi H-7600, Japan), respectively. Fluorescence was detected with confocal laser scanning microscopy (CLSM, Leica TCS‐SP2, German), and the size distribution and zeta potential were determined using a Malvern Zetasizer Nano ZS (Malvern Instruments, UK). Absorption spectrum of microcapsules was detected with a Lambda 950 ultraviolet spectrophotometer (PerkinElmer Lambda 950, USA).

### Encapsulation efficiency and drug-loading efficiency of HMME/PLGA microcapsules

Encapsulation efficiency and drug-loading efficiency of the HMME/PLGA microcapsules were determined using the ultraviolet (UV) spectrophotometric method. The encapsulation efficiency and drug-loading efficiency were calculated using the following formulations^32^,^33^,^34^,^35^: Encapsulation efficiency (EE):(EE%) = (W_i_/C_t_) × 100%, and Loading efficiency (LE):(LE%) = (W_i_/W_T_) × 100%, where W_i_ was the total drug amount in the HMME/PLGA microcapsules, C_t_ was the total weight of HMME used in the microcapsule preparation, and W_T_ was the total weight of HMME/PLGA microcapsules.

### Cell line and cell culture

Ovarian cancer cell line SKOV_3_ was obtained from the Chongqing Key Laboratory of Ultrasound Molecular Imaging of Chongqing Medical University. Cells were cultured with complete RPMI medium (Shanghai source leaf Biological Technology Co., Ltd., China), supplemented with 100 mg/ml penicillin (Nanjing Oddo foni Biology Technology Co. Ltd., China), 100 mg/ml streptomycin (North China Pharmaceutical Limited by Share Ltd., China), and 10% fetal bovine serum (Shanghai Chuan Xiang Biological Technology Co., Ltd., China) in a 37 °C, 5% CO^2^ incubator.

### Animals and model establishment

Totally 60 Female BALB/c nude mice (strain nu/nu), 4–6 weeks old, weighing 15–22 g, were purchased from the Experimental Animal Center of Chongqing Medical University. All animal experimental protocols were reviewed and approved by the Chongqing Medical University Animal Care Committee. The methods were carried out in accordance with the approved guidelines. The mice were housed under a constant temperature and humidity condition. For the tumor model establishment, each nude mouse was subcutaneously inoculated with 1 × 10^6^ SKOV_3_ cells in 100 μl serum-free RPMI-1640 medium, in the right flank.

### Cell toxicity-proliferation test

SKOV_3_ cells in the logarithmic phase were used to make cell suspension of 5 × 10^4^ cells/ml. These cells were randomly divided into the following groups: the control group (Group I), the group subjected to US (US; Group II), the group treated with PLGA subjected to US (PLGA+US; Group III), the group treated with 20 μg/ml HMME subjected to US (HMME+US; Group IV), and the group treated with HMME (20 μg/ml)/PLGA microcapsules subjected to US (HMME/PLGA+US; Group V). 2 ml cell suspension from Groups II-V, respectively, was added into a 50-ml centrifuge tube with an acoustic passing film bottom. These cells were subjected to irradiation with the CGZZ US gene transfection apparatus (Chongqing Haifu Medical Technology Co., Ltd., China) from the bottom, at the intensity of 0.50 w/cm^2^ and frequency of 1 MHz, for 10, 30, 60, and 90 s, respectively. The control group (Group I) received no treatment. After the US irradiation, 100 μl cell suspension was planted onto a 96-well culture plate. 10 μl CCK-8 reagent (Yiyuan Biotechnology Co., Ltd., China) was added into each well to incubate the cells at 37 °C for 3–4 h. The optical density (OD) at 490 nm was read with the EL × 800 Universal Microplate Reader (Bio-Tek Instrument Inc., USA). Experiments were repeated three times. Cell viability rate was calculated according to the following formation: Cell viability rate (%) = (OD_treatment_−OD_blank_/OD_control_−OD_blank_) × 100%.

Based on results from the above experiments, irradiation duration was set as 10 s, with the same US irradiation parameters and HMME concentrations. After treatments, cell suspension was planted onto another 96-well culture plate, and incubated for 12, 24, 36, and 48 h, respectively. After incubated with the CCK-8 reagent, OD at 490 nm was read. Experiments were repeated three times.

### *In vitro* US and PA dual-modality imaging

US imaging was assessed with the gel mold having holes (2 cm in depth) on the edge. The holes were filled with PBS, PLGA, HMME, and HMME/PLGA microcapsules with different HMME concentrations (0.25, 0.50, and 1.00 mg/ml) to obtain the US imaging results of these samples. All images were acquired using a 21 MHz linear-array ultrasound transducer (VIVO 2100; FUJIFILM Visual Sonics, Inc., Canada) with the conventional B mode, with the same instrument parameters. Mean echo intensity (Mean DB) was calculated by the DFY software (Institute of Ultrasound Imaging of Chongqing Medical Sciences, China). For the *in vitro* PA imaging, PBS, PLGA, HMME, and HMME/PLGA microcapsules were subjected to the laser exposure at 690 nm for about 3 min, and the PA intensity variation was observed using the VEVO LASR PA imaging system. Experiments were repeated three times.

### *In vivo* US and PA dual-modality imaging

*In vivo* US and PA dual-modality imaging was performed about one month after cell inoculation, when the tumor sizes reached 8–10 mm. To investigate the enhancing effect of HMME/PLGA microcapsules on US and PA imaging, totally 10 mice with detectable ovarian cancer were anesthetized, and intravenously injected with 0.2 ml HMME/PLGA microcapsule solution (the HMME concentration was 1.50 mg/ml), followed by the laser exposure at 690 nm. For 5 mice, PA imaging for tumors was performed at 0, 30, 60, 90, 120, and 150 min after irradiation. Corresponding US images were recorded at corresponding time points for the other 5 mice. PA average value and US average gray scale of the tumors were detected using the VEVO LASR PA imaging system and the DFY software, respectively. Experiments were repeated three times.

### *In vitro* HIFU exposure

*In vitro* HIFU exposure was performed according to following protocols. Fresh bovine (Chongqing Jiangbei cattle and sheep Muslim Market, China) (10 cm × 10 cm × 8 cm) was placed in a container immersed in degassed water after warming. The sample was divided into the following groups: PBS (Group I), PLGA (Group II), HMME (Group III), and HMME/PLGA microcapsule (Group IV) groups (C_HMME_ = 1.50 mg/ml). HIFU ablation was performed with single irradiation, with output acoustic powers of 120, 150, and 180 w, respectively. For each output acoustic power, the treatment duration was set as 3, 5, and 10 s, respectively. Gray scale values were recorded in the ablation areas before and after ablation, using the Gray Val 1.0 software (Chongqing Haifu Medical Technology Co., Ltd., China). After irradiation, the coagulative necrosis volume (V) and energy efficiency factor (EEF) were calculated according to the following formulations: V (mm^3^) = (π/6) × length × width × depth; EEF (J/mm^3^) = ηPt/V^36^, where η was the focusing coefficient of HIFU transducer (in this instrument η was set as 0.7), P (W) was the total acoustic power of HIFU, and t (s) was the total treatment time. EEF represents the US energy required to damage the unit volume of tumor or other lesions.

### *In vivo* HIFU exposure

About 30 days after the tumor inoculation, 50 nude mice bearing xenograft tumors were randomly divided into the control (Group I), PBS (Group II), PLGA (Group III), HMME (Group IV), and HMME/PLGA microcapsule (Group V) groups. Nude mice were anesthetized just before ablation. Group I only received HIFU ablation. For Groups II-V, the nude mice received injection of 200 μl PBS, PLGA, HMME, and HMME/PLGA microcapsule (C_HMME_ = 1.50 mg/ml), respectively, at the tumor sites, which were massaged for 3 min before HIFU exposure. The nude mice were placed on the HIFU treatment bed in a prone position, with the tumor sites completely immersed in degassed water. For all the five groups, each tumor was destroyed by one single exposure^37^, with the acoustic power at 120 w and exposure duration for 5 s. During HIFU treatment, the ablation effects were assessed using diagnostic ultrasonic imaging in real time. The gray scale values of the targeted areas before and after ablation were recorded and compared by the Gray Val 1.0 software affiliated to the HIFU equipment.

### Histopathological detection

At 1 h after treatment and detection, the nude mice were anesthetized and the tumors were removed immediately. After sectioned into slices, the maximal section of necrotic tumor tissue was selected for staining with TTC solution for 30 min. Histopathological analysis was performed for the ablated and the surrounding tissues from each tumor with the hematoxylin and eosin (HE) staining.

### Immunohistochemisty

Cell proliferation and apoptosis process in the target tissue were detected with the proliferating cell nuclear antigen (PCNA) and TdT-mediated dUTP nick end labeling (TUNEL) methods. Proliferating index (PI) and apoptotic index (AI) were expressed as the ratio of positively stained tumor cells out of the total cells, which were determined from 5 random fields at 400× magnification. Target tissues were sampled by glutaraldehyde to observe the ultrastructural changes with TEM. The volume of coagulative necrosis and EEF in the target area were calculated as mentioned above.

### Statistical analysis

Data were expressed as mean ± SD. One way ANOVA was performed for multiple comparison, and student’s t-test were used for intergroup comparison. *P* < 0.05 was considered as statistically significant.

## Additional Information

**How to cite this article**: Yan, S. *et al*. HematoPorphyrin Monomethyl Ether polymer contrast agent for ultrasound/photoacoustic dual-modality imaging-guided synergistic high intensity focused ultrasound (HIFU) therapy. *Sci. Rep.* **6**, 31833; doi: 10.1038/srep31833 (2016).

## Supplementary Material

Supplementary Information

## Figures and Tables

**Figure 1:**
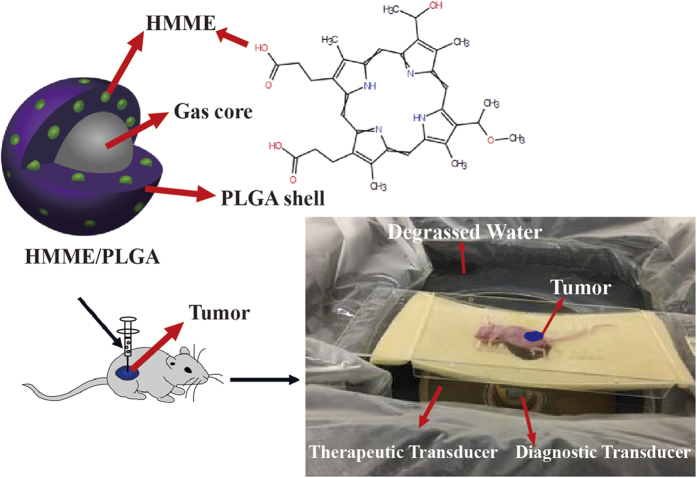
Schematic illustration of the microstructure of HMME/PLGA microcapsules and the progress of introduction of these microcapsules into HIFU cancer surgery.

**Table 1:** The effect of ultrasound irradiation on the survival rate of SKOV_3_ cell at different duration of Group V.

Time	N	OD value	Survival rate (%)
0 s	10	0.6115 ± 0.0225	100%
10 s	10	0.4190 ± 0.0192*	60.1%
30 s	10	0.3973 ± 0.0132*	55.6%
60 s	10	0.3210 ± 0.0191*,#	39.7%
90 s	10	0.3155 ± 0.0070*,#	38.6%

Values are mean ± SD.

^*^p < 0.05 vs 0 s.

^#^*p* < 0.05 vs other groups.
