# Supplementary material for: HematoPorphyrin Monomethyl Ether polymer contrast agent for ultrasound/photoacoustic dual-modality imaging-guided synergistic high intensity focused ultrasound (HIFU) therapy
Source: Sci Rep. 2016 Aug 18;6:31833. doi: 10.1038/srep31833 (PMC4989155; doi:10.1038/srep31833)
Supplement: Supplementary Information [file srep31833-s1.doc]

**HematoPorphyrin Monomethyl Ether polymer contrast agent for ultrasound/photoacoustic dual-modality imaging-guided synergistic high intensity focused ultrasound (HIFU) therapy**

**Sijing Yan^1＃^, Min LU^2＃^, Xiaoya Ding^1^, Fei Chen^1^, Xuemei He^3^, Chunyan Xu^4^, Hang Zhou^3^, Qi Wang^1^, Lan Hao^4﹡^, ＆ Jianzhong Zou^1﹡^**

^1^State Key Laboratory of Ultrasound Engineering in Medicine Co-Founded by Chongqing and the Ministry of Science and Technology, Chongqing Key Laboratory of Biomedical Engineering, College of Biomedical Engineering, Chongqing Medical University, Chongqing 400016, P.R. China

^2^Ultrasound department of Second Hospital Affiliated to Chongqing Medical University, Institute of Ultrasound Imaging, Chongqing Medical University, Chongqing 400010, P.R. China

^3^Ultrasound department of First Hospital Affiliated to Chongqing Medical University, Chongqing 400016, P.R. China

^4^Second Affiliated Hospital of Chongqing Medical University & Chongqing Key Laboratory of Ultrasound Molecular Imaging 400016, P.R. China

**Additional information**


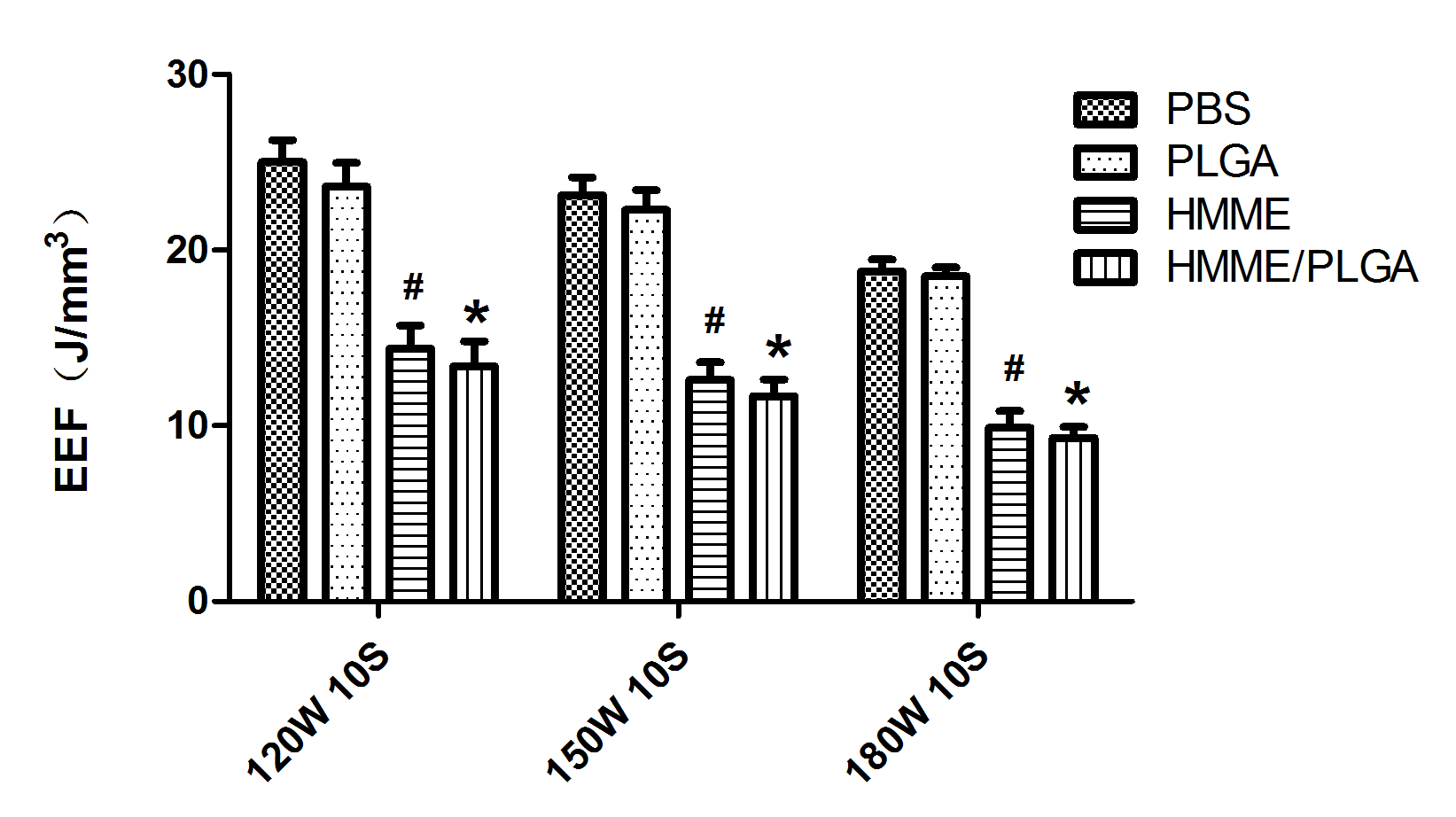


**Supplementary Figure S1.** The EEF value of the four groups after HIFU ablation *in*  *vitro*.**^＊^***P*＜0.05 vs the other groups; ^＃^*P*＜0.05 vs PBS and PLGA groups.
